# To be or not to be hallucinating: Implications of hypnagogic/hypnopompic experiences and lucid dreaming for brain disorders

**DOI:** 10.1093/pnasnexus/pgad442

**Published:** 2023-12-19

**Authors:** Guglielmo Foffani

**Affiliations:** HM CINAC (Centro Integral de Neurociencias Abarca Campal), Hospital Universitario HM Puerta del Sur, HM Hospitales, Madrid 28938, Spain; Hospital Nacional de Parapléjicos, Toledo 45004, Spain; CIBERNED, Instituto de Salud Carlos III, Madrid 28031, Spain

**Keywords:** dream lucidity, neurology, psychiatry, mental health, hallucinations, hypnagogic, hypnopompic

## Abstract

The boundaries between waking and sleeping—when falling asleep (hypnagogic) or waking up (hypnopompic)—can be challenging for our ability to monitor and interpret reality. Without proper understanding, bizarre but relatively normal hypnagogic/hypnopompic experiences can be misinterpreted as psychotic hallucinations (occurring, by definition, in the fully awake state), potentially leading to stigma and misdiagnosis in clinical contexts and to misconception and bias in research contexts. This Perspective proposes that conceptual and practical understanding for differentiating hallucinations from hypnagogic/hypnopompic experiences may be offered by lucid dreaming, the state in which one is aware of dreaming while sleeping. I first introduce a possible systematization of the phenomenological range of hypnagogic/hypnopompic experiences that can occur in the transition from awake to REM dreaming (including hypnagogic perceptions, transition symptoms, sleep paralysis, false awakenings, and out-of-body experiences). I then outline how metacognitive strategies used by lucid dreamers to gain/confirm oneiric lucidity could be tested for better differentiating hypnagogic/hypnopompic experiences from hallucinations. The relevance of hypnagogic/hypnopompic experiences and lucid dreaming is analyzed for schizophrenia and narcolepsy, and discussed for neurodegenerative diseases, particularly Lewy-body disorders (i.e. Parkinson's disease, Parkinson's disease dementia, and dementia with Lewy bodies), offering testable hypotheses for empirical investigation. Finally, emotionally positive lucid dreams triggered or enhanced by training/induction strategies or by a pathological process may have intrinsic therapeutic value if properly recognized and guided. The overall intention is to raise awareness and foster further research about the possible diagnostic, prognostic, and therapeutic implications of hypnagogic/hypnopompic experiences and lucid dreaming for brain disorders.

## Introduction

The transition between wakefulness and sleep, whether falling asleep (hypnagogic) or waking up (hypnopompic), is a gateway of experiences that has always intrigued humanity. Hypnagogic/hypnopompic experiences are not defined in an ontological sense by what they are, but in a temporal sense by when they occur. Somehow related to ordinary dreaming, but with higher levels of awareness, hypnagogic/hypnopompic experiences can involve different sensory modalities and display a wide phenomenological richness: from vague to vivid, from simple to complex, from mundane to bizarre, from terrifying to blissful (1–3). This richness is likely caused by relatively direct transitions from waking to REM dreaming, which can occur sometimes, particularly in the last cycles of sleep toward the end of the night (4, 5). Sleep-onset REM periods were originally described in patients with narcolepsy (4), but can also be observed in the normal general population (6, 7). The profound impact of florid hypnagogic/hypnopompic experiences on culture and collective imagery can be traced in religion (8), folklore (9–11), and art (12, 13). Even though most hypnagogic/hypnopompic experiences are relatively normal, they can be challenging for our ability to monitor and interpret reality.

Dreaming notoriously shares some similarities with psychotic states (14). Consequently, normal dreaming-related hypnagogic/hypnopompic experiences, possibly enhanced by pathological processes and/or by therapeutic drugs, can be phenomenologically confounded with psychotic hallucinations, which belong by definition to the fully awake state. This confusion may happen from very rarely to rather frequently depending on sociocultural factors that are difficult to ascertain, since formal studies are not available. In clinical contexts, misinterpreting hypnagogic/hypnopompic experiences as hallucinations may lead to misdiagnosis, mistreatment, and stigma (15–19). In research contexts, the misinterpretation may produce misconception and bias. This Perspective proposes that conceptual and practical understanding for better differentiating hallucinations from hypnagogic/hypnopompic experiences may be offered by lucid dreaming, the “non-pathological variant of normal REM dreaming, in which one is aware of dreaming while continuing to sleep” (20).

The Perspective will analyze the key sources of possible misinterpretation between hypnagogic/hypnopompic experiences and hallucinations. I will propose a systematization of the phenomenological range and temporal sequence of relatively normal hypnagogic/hypnopompic experiences that can occur in the transition from awake to REM dreaming. I will then suggest that lucid dreaming offers a novel perspective on how individuals may learn to test and probe the difference between hallucinations and hypnagogic/hypnopompic experiences, and potentially play a role in positively reinterpreting those experiences. The relevance of this differentiation and its relationship with lucid dreaming will then be analyzed from the viewpoint of two representative brain disorders: schizophrenia, the paradigmatic disease for psychotic hallucinations, and narcolepsy, the paradigmatic disease for hypnagogic/hypnopompic experiences. The distinction between schizophrenia and narcolepsy offers a conceptual framework that will then be discussed for neurodegenerative diseases and particularly for Lewy body disorders (i.e. Parkinson's disease, Parkinson's disease dementia, and dementia with Lewy bodies), where putative hallucinations are likely a mix of genuine psychotic hallucinations (21–23) and nonpsychotic, hypnagogic/hypnopompic experiences (24–26). Finally, the intrinsic therapeutic value of lucid dreaming for brain disorders will be discussed. In order to make the balance between evidence and speculation as explicit as possible, key testable hypotheses and corresponding current levels of evidence will be highlighted and summarized in Table 1.

**Table 1.** Testable hypotheses and corresponding current level of evidence.

Testable hypotheses	Definitive evidence	Preliminary evidence	Hypothesis
1. Hypnagogic/hypnopompic experiences may be erroneously rated as hallucinations in clinical scales			X
2. Updated scales rating both hallucinations and hypnagogic/hypnopompic experiences would increase diagnostic sensitivity and specificity			X
3. Metacognitive strategies (i.e. the “third person” strategy and the “reality check” strategy) may be useful for some patients to differentiate between hallucinations and hypnagogic/hypnopompic experiences			X
4. Lucid dreaming frequency may be unchanged while dream control may be increased in psychosis/schizophrenia		X	
5. Insight in dreaming and in psychosis may rely on different cognitive/metacognitive mechanisms		X	
6. Lucid dreaming frequency is increased in narcolepsy	X		
7. In Lewy body disorders, hallucinations have prognostic value for faster development of dementia	X		
8. In Lewy body disorders, hypnagogic/hypnopompic experiences may represent a more favorable prognosis compared to hallucinations		X	
9. Lucid dreaming frequency may correlate with hypnagogic/hypnopompic experiences			X
10. Lucid dreaming frequency may have prognostic value			X
11. Lucid dreaming may have extrinsic therapeutic value for treating nightmare disorder		X	
12. Lucid dreaming may have intrinsic therapeutic value for personal fulfillment		X	

## Misinterpretation of hypnagogic/hypnopompic experiences as hallucinations

According to the Diagnostic and Statistical Manual of Mental Disorders fifth edition (DSM-5), a hallucination is “a perception-like experience with the clarity and impact of a true perception but without the external stimulation of the relevant sensory organ”. The DSM-5 specifies that “the term hallucination is not ordinarily applied to the false perceptions that occur during dreaming, while falling asleep (hypnagogic), or upon awakening (hypnopompic)”. In other words, to experience genuine hallucinations, one should be fully awake and, reciprocally, hypnagogic/hypnopompic experiences are not hallucinations. This very definitional aspect, often disregarded in the literature, provides the basis for analyzing the key sources of possible misinterpretation between hypnagogic/hypnopompic experiences and hallucinations as well as for proposing possible solutions.

The first source of misinterpretation is semantic, arising from ambiguous terminology combined with possible lack of knowledge. The oxymoronic expression “hypnagogic hallucinations”, commonly used in the literature (including the DSM-5 when describing narcolepsy), is a semantic inconsistency in which the adjective (hypnagogic) changes the meaning of the noun (hallucinations). Citing the foreword of the DMS-II, published in 1968, “rationalists may be prone to believe the old saying that ‘a rose by any other name would smell as sweet’; but psychiatrists know full well that irrational factors belie its validity and that labels of themselves condition our perceptions”. The wordings “hypnagogic hallucinations”, “hypnopompic hallucinations”, “sleep related hallucinations” and similar variants should thus probably be reconsidered. Furthermore, clinical scales currently available to assess hallucinations typically do not include any item for hypnagogic/hypnopompic experiences (Table 2). This is not incorrect from a purely theoretical standpoint, but in clinical and research practice it can lead to response bias (i.e. hypnagogic/hypnopompic experiences rated as hallucinations), unless the differentiation is totally clear to the subject/rater, which is often not the case (hypothesis 1, Table 1). Updated scales that rate both hallucinations and hypnagogic/hypnopompic experiences would help make the differentiation explicit, thus increasing diagnostic sensitivity and specificity and reducing ambiguity in the literature (hypothesis 2, Table 1).

**Table 2.** Clinical scales commonly employed to screen or assess hallucinations.

Scale	Hallucinations (num. items)	Hypnagogic/hypnopompic experiences (num. items)
Clinician-Rated Dimensions of Psychosis Symptom Severity (CRDPSS)	1	0
Brief Psychiatric Rating Scale (BPRS)	1	0
Neuropsychiatric Inventory (NPI)	1	0
Neuropsychiatric Inventory–Questionnaire (NPI-Q)	1	0
Positive and Negative Syndrome Scale for Schizophrenia (PANSS)	1	0
Scale for the Assessment of Positive Symptoms (SAPS)	7	0
Launay-Slade Hallucination Scale (LSHS)	12	0
Revised Hallucination Scale (RHS)	16	0
Cardiff Anomalous Perception Scale (CAPS)	32	0
Movement Disorders Society-Unified Parkinson's Disease Rating Scale (MDS-UPDRS)	1	0

The second source of misinterpretation is perceptual. Reality monitoring, i.e. our ability to discriminate between the external and internal sources of perceptual experiences, has at least two levels: sensory and cognitive (27). The sensory level is what allows us to *experience* something as real, whereas the cognitive level is what allows us to *know* whether what we experience has an external or internal origin (27). When the strength of a signal in first-order sensory processes reaches a sort of “reality threshold”, it is perceived as real independently of its internal or external origin (28). Most internally generated experiences, such as memories or imaginations, simply do not reach the perceptual reality threshold, while most externally generated experiences do. Consequently, cognitive reality monitoring is in most cases a relatively straightforward threshold-based discrimination. Problems and challenges may arise when internally generated experiences do reach the perceptual reality threshold (28), as is the case for hallucinations, dreams, and many hypnagogic/hypnopompic experiences. Hypnagogic/hypnopompic experiences that reach the perceptual reality threshold may be indistinguishable from hallucinations.

Possible perceptual misinterpretations can occur in the well-known hypnagogic/hypnopompic state of sleep paralysis, a REM parasomnia that is spontaneously experienced in isolated or recurrent form by about 8% of the general population (with estimates ranging from 2 to 60%) and is particularly prevalent in patients with nightmare disorder, post-traumatic stress disorder, and anxiety disorders (29, 30). Sleep paralysis offers an example of how a sense of being awake is not sufficient to infer that one is necessarily awake. In fact, recent polysomnographic findings suggest that during sleep paralysis the brain is not in an awake state, as classically considered, but in a dreaming state (31). This possibly plays a role in the explanation of a number of apparently paranormal experiences (29), as well as in the highly distressful incubus phenomenon and other “hallucinations” that are frequently associated with sleep paralysis (11, 32): since emotions populate the dreaming reality in a very tangible way, the distress is likely causing the incubus and the “hallucinations” at least as much as the other way around. Because they do not occur in the fully awake state, sleep paralysis “hallucinations” are, by definition, not hallucinations but hypnagogic/hypnopompic experiences.

Even more prone to perceptual misinterpretation is a lesser known hypnagogic/hypnopompic state that can be defined as realistic dreaming, which is hardly acknowledged in the psychological/psychiatric literature (33, 34). Realistic dreaming does not refer here to mundane, non-surreal dream content, but more specifically to a state in which the dream content has a high overlap/similarity with the preceding waking situation (similar to sleep paralysis but without the sensation of paralysis). Intuitively, the temporal proximity and possible continuity of awareness from awake to REM dreaming can produce a perceptual continuity between waking and dreaming. Consequently, the dreaming scene may seem identical to waking reality in a very convincing way. The apparently literal scene, perceived either subjectively as a false awakening (31) or autoscopically as an out-of-body experience (35), may then become enriched with more bizarre dreaming elements (e.g. things and beings that appear distorted or are not supposed to be there), which appear very much like psychotic illusions and hallucinations. Significant events occurring in a misinterpreted realistic dreaming state can then be erroneously remembered as having occurred in waking reality, becoming delusional memories. Realistic dreaming may thus induce anoneirognosis, i.e. dream–reality confusion (36), even in absence of pathological alterations affecting the mechanisms of reality testing.

To avoid the perceptual misinterpretation of hypnagogic/hypnopompic experiences as hallucinations, it is necessary to introduce a third level of reality testing that is neither sensory nor cognitive but more broadly metacognitive (27). At this level, the issue is not about discriminating between the external or internal sources of perceptual experiences, but about discriminating the awake or dreaming state in which those experiences occur. This is exactly what happens in lucid dreaming. The central hypothesis put forward in this Perspective is thus that lucid dreaming may offer conceptual and practical tools for differentiating hallucinations from hypnagogic/hypnopompic experiences.

## Systematization of hypnagogic–hypnopompic experiences from wake-initiated lucid dreams

Ordinary non-lucid dreaming is a delusional state of normal sleeping, in the sense that the non-lucid dreamer maintains—at least while sleeping—the false belief of being in a non-dreaming reality despite the contradictory evidence provided by the inconsistency and bizarreness of oneiric percepts. In other words, non-lucid dreaming lacks the metacognitive level of reality testing.

Lucid dreaming is instead a less common non-delusional state of normal sleeping, in which the dreamer is aware of dreaming while sleeping. Metacognitive reality testing is thus the definitional aspect of lucid dreaming. Even though the concept goes back thousands of years (37), lucid dreaming officially entered the neuroscientific literature about four decades ago, when dream lucidity was documented polysomnographically (38, 39), and has recently gained scholarly attention (39–45). In the general population, lucid dreaming is not exceedingly rare: when explicitly asked, about 55% of people report having had at least one lucid dream in a lifetime, and about 23% of people have at least one lucid dream per month (46). However, in neurology and psychiatry, lucid dreaming remains a largely understudied or completely unknown phenomenon, possibly because its clinical implications and significance are still unclear.

Compared to non-lucid dreams, lucid dreams display higher levels not only of lucid insight, but also of control over thoughts and actions, of logical thinking, of memory access to elements of waking life, of dissociative experience, and of positive emotions (41, 47). These cognitive/metacognitive features correspond to increased activity and connectivity among several regions in fronto-parietal cortical networks during lucid compared to non-lucid REM sleep (39, 48, 49) and during thought monitoring in subjects with high dream lucidity compared to subjects with low dream lucidity (43). Lucid dreams can occur spontaneously, particularly in children, and their frequency decreases through adolescence (50) but can be increased with training at any age (51–53). Even though in the nonscientific literature lucid dreams may be described as part of a more complex phenomenology of related experiences, here the term “lucid dreaming” is used as a synecdoche (i.e. the part for the whole) to refer to any lucid experience occurring while sleeping or at its boundaries (i.e. including the state of realistic dreaming defined above).

**Fig. 1.**
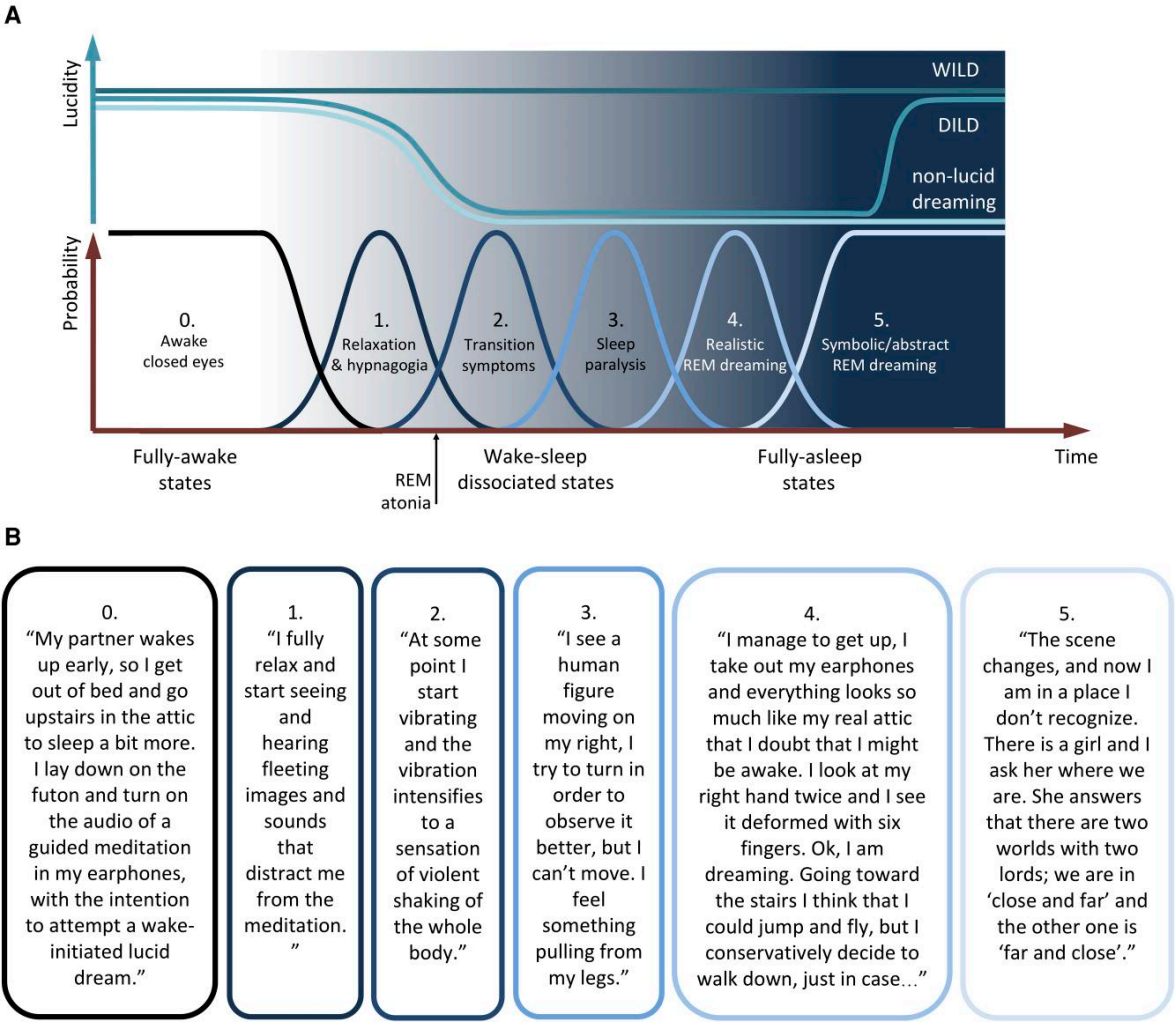
Range of normal hypnagogic experiences associated with wake-to-REM transitions. (A) Schematic representation of the main psychophysiological states that can be experienced in wake-initiated lucid dreaming (WILD) compared to dream-initiated lucid dreaming (DILD) and ordinary non-lucid dreaming through a continuum of states from fully awake to fully asleep. The upper part represents a simplified temporal evolution of lucidity. The lower part represents the proposed sequence of states in terms of probability of being in a given state, with possible overlap between states. Note that the drawing offers a simplified version of a process that is admittedly complex and subjectively variable. These states are not necessarily all experienced (or remembered) in a typical WILD. Similar states can also be consciously experienced in the hypnopompic transition from a lucid dream to waking reality. (B) A representative dream report of an intentional WILD in a normal subject, with examples of subjective experiences corresponding to each of the different states represented in (A).

In terms of initiation dynamics, there are two main types of lucid dreams (54, 55): dream-initiated lucid dreams, which are more common, and wake-initiated lucid dreams, which are less common but more relevant for the present narrative (Fig. 1A). In a dream-initiated lucid dream, the dreamer acquires lucidity at some point within a dream: in a sort of eureka moment, the dreamer recognizes that some inconsistency or bizarreness of the oneiric percept is actually oneiric, and a discrete shift occurs from non-lucidity to lucidity. In a wake-initiated lucid dream, conversely, the subject learns to maintain lucidity while transitioning from the waking state to the REM dreaming state. This is a sort of lucid variant of the classical sleep-onset REM periods (4, 6, 7). A wake-initiated lucid dream is typically easier to achieve after a nocturnal awakening toward the end of sleep (54, 55), when waking and REM sleeping are physiologically closer, and the occurrence probability of sleep-onset REM periods is higher (4, 5). Intermediate levels of oneiric awareness are also possible and common.

The experience of wake-initiated lucid dreams provides empirical knowledge about different psychophysiological states that characterize the transition from waking to REM dreaming. Based on subjective reports and personal experience, and acknowledging some simplification of a process that is admittedly complex and subjectively variable, at least five common states can be encountered in a lucid wake-to-REM transition, offering the following possible systematization (Fig. 1B). First there is an initial state of body relaxation, possibly with hypnagogic perceptions, which is experienced by the majority of the population at sleep onset (56). Second, transition symptoms may appear (e.g. body distortions such as vibrations, “electrical” sensations, loud rushing sounds, etc.) (35), which presumably reflect the changes in brain signaling associated with the onset of REM sleep atonia, i.e. the diffuse reduction of muscle tone that physiologically prevents the enactment of dreams. Transition symptoms can be typically differentiated from the initial hypnagogic perceptions due to their nonvisual predominance and higher level of realism. The mechanisms underlying this mix of somatosensory and auditory sensations are not clear, but anatomical and functional links between feeling and hearing (57–59) may play a role for these internally generated perceptions. Loud and abrupt auditory transition symptoms may be the phenomenological basis of the exploding head syndrome (60), a benign parasomnia “characterized by a sudden, loud imagined noise or sense of a violent explosion in the head occurring as the patient is falling asleep or waking during the night” (61). The third state is sleep paralysis, which is the physiological equivalent of the well-known REM parasomnia, and may include the canonical sense of being paralyzed combined with more or less complex visual/auditory percepts (29, 62, 63). Sleep paralysis is then followed by the entry into the state of realistic dreaming previously discussed, perceived either as a false awakening (31, 64) or as an out-of-body experience (35), in which the sense of being paralyzed is lost and the dreaming reality looks and feels exactly the same as the waking reality. Finally, there is a transit toward a more complex state of “symbolic/abstract” REM dreaming, more similar to the lucid oneiric experience of a dream-initiated lucid dream (41, 54, 55). These states are not necessarily all experienced (or remembered) in a typical wake-initiated lucid dream, nor their boundaries always evident, but they can be at least partly characterized polysomnographically (31, 54, 63, 65, 66). Similar states can also be consciously experienced in the hypnopompic transition from a lucid dream to waking reality. Spontaneous occurrence of intermediate states, if recognized, can also be used as a sort of shortcut toward lucid dreaming. This is particularly the case for spontaneous sleep paralysis, which lucid dreamers often recognize and use—stripped of any negative emotional content (67)—as a gateway toward lucid dreaming states (68).

The dynamics of lucid dreaming thus somehow systematize the range of hypnagogic/hypnopompic experiences classically described since the pre-lucid-dreaming literature (1) and clarify that some hybrid/dissociated sleep–wake states often attributed to pathological conditions (69), such as sleep paralysis (29, 62) and out-of-body experiences (70–73), are also part of the normal hypnagogic/hypnopompic continuum (74, 75).

## Metacognitive strategies for differentiating hallucinations against hypnagogic/hypnopompic experiences

Lucid dreaming may also offer practical understanding for differentiating hypnagogic/hypnopompic experiences from hallucinations. Specifically, the metacognitive reality testing that characterizes dream lucidity may be crucial not only to escape the delusional state of ordinary dreaming, but also to recognize that bizarre experiences occurring in non-awake states are not hallucinations but relatively normal nonpsychotic experiences. Metacognitive strategies of reality testing would thus be useful, both for clinical and research purposes, to avoid the perceptual misinterpretation of hypnagogic/hypnopompic experiences as hallucinations (hypothesis 3, Table 1). Two simple strategies for practically differentiating the two phenomena may be formally tested in future investigations.

The first strategy is to confirm hallucinations by seeking evidence of being awake while hallucinating. This evidence may be achieved—and indeed often is—by simply describing the hallucinations to a third subject (“dear, do you see that giraffe that I am seeing?”). Crucially, the third person should later confirm that the communication has actually taken place (and that the subject was not sleep-talking). The least ambiguous implementation of this first strategy is when a patient describes to his/her doctor a hallucination while hallucinating, which happens often in the clinic.

The second strategy is to confirm hypnagogic/hypnopompic experiences by seeking evidence of being dreaming rather than fully awake. This evidence may be achieved by performing a “reality check”, which is a simple metacognitive maneuver commonly employed by lucid dreamers to differentiate dreaming reality from waking reality. A reality check is the combination of a question about the state of reality (i.e. “am I dreaming?”) followed by a volitional action that is “impossible” to perform in waking reality (e.g. pass a finger through the palm of the other hand) and/or by an observation that would be expected in waking reality but is very unlikely in the more unstable dreaming reality (e.g. watching the hands twice and counting the same number of fingers). If the impossible action is achieved (e.g. the finger passes through the palm) or the unlikely observation is rejected (e.g. the hands have more or less than ten fingers), then one attains confirmation of being dreaming (and thus not hallucinating). Note that this confirmation also transforms the dream into a lucid dream.

The “third-person” strategy is somewhat more objective, but only allows confirming the awake state. The “reality-check” strategy is more subjective, but allows confirming both the awake and the dreaming states. Important caveats are that both strategies may fail (more probably in cognitively impaired patients), that caution may well be warranted before encouraging training in lucid dreaming (76, 77), and that the dualistic split of awake vs. dreaming is a useful simplification, but it may be admittedly too simplistic to capture the full complexity of dissociated sleep–wake states. With the fast development of wearable technologies for sleep–wake scoring (78), the ideal polysomnographic confirmation of hallucinations vs. hypnagogic/hypnopompic experiences (25) may become clinically viable in the home setting, at least for some patients. In any case, common accounts of supposed hallucinations occurring around sleep periods in brain disorders await to be confirmed as genuine hallucinations or converted into lucid dreams.

## Hallucinations, hypnagogic/hypnopompic experiences, and lucid dreaming in schizophrenia vs. narcolepsy

The prototypic brain disorder for hallucinations is schizophrenia. Hallucinations in schizophrenia are most typically auditory, but they are also common in the visual and other sensory modalities (79). Similarly to dreams, psychotic hallucinations may be lucid or non-lucid, depending on the level of insight of the patients about the nature of their hallucinations (80, 81). Despite the long-observed similarities between psychotic states and dreaming (14), only one study has explicitly investigated lucid dreaming in schizophrenia patients (and in psychotic patients with a diagnosis of bipolar disorder), who unexpectedly displayed greater ability to control their lucid dreams and essentially normal lucid dreaming frequency (82) (hypothesis 4, Table 1). Furthermore, in contrast with the view that insight in dreaming and psychosis may share some neural correlates (80), psychotic patients that were more lucid about their dreams were *not* more lucid about their hallucinations (82). Lucid dreaming and hallucinations with insight may thus rely on different cognitive/metacognitive mechanisms (hypothesis 5, Table 1). Nevertheless, the authors themselves refer to their results as preliminary (82), indicating the need for further studies on lucid dreaming in schizophrenia/psychosis and on the multifaceted relationships between insight and metacognition in health and disease (83).

At the other extreme, the prototypic brain disorder for hypnagogic/hypnopompic experiences is narcolepsy, which is characterized by excessive daytime sleepiness, cataplexy, hallucination-like hypnagogic/hypnopompic perceptions, sleep paralysis, and disrupted nocturnal sleep (including REM sleep behavior disorder) (84). These features are likely caused by the neurodegeneration of hypocretin/orexin neurons in the hypothalamus (85, 86). These neurons crucially contribute to promote wakefulness in the circadian regulation of the sleep/wake cycle, and in the stabilization of REM sleep (87). Problems (and possible solutions) in the differential diagnosis of narcolepsy vs. schizophrenia have a long history in psychiatry (16, 88–91), but the nonpsychotic non-hallucinatory nature of the bizarre experiences reported by narcoleptics is relatively well established (88), although not always recognized. The great majority of presumed hallucinations reported by narcoleptics—besides relatively rare comorbid psychosis and more common iatrogenic psychotic symptoms triggered by psychostimulants—are in fact hypnagogic/hypnopompic experiences (88). These experiences may be explained, at least in part, by the high prevalence and occurrence of sleep-onset REM periods in narcolepsy (4, 92). Narcoleptics thus spontaneously experience, with high frequency and varying degree of awareness, the full range of hybrid/dissociated states that are volitionally sought by lucid dreamers in wake-initiated lucid dreams (Fig. 1) (69). In agreement with the notion that wake-REM sleep transitions may be instrumental for dream lucidity (93), in narcoleptics the average frequency of lucid dreaming is at least one order of magnitude higher than in the normal population (94, 95) (hypothesis 6, Table 1). Despite their nonpsychotic and non-hallucinatory nature, vividly realistic dreaming experiences in narcoleptics can nevertheless induce anoneirognostic delusional confusion of dreamed events with waking reality (96), posing challenges in daily life and clinical practice (88). On the other hand, the natural ability of narcoleptics for experiencing hybrid/dissociated states and lucid dreaming is possibly connected with higher creative thinking (97).

The dichotomy between genuine hallucinations associated with presumably normal lucid dreaming frequency in schizophrenia and hypnagogic/hypnopompic experiences associated with higher lucid dreaming frequency in narcolepsy seems attractive from a diagnostic and semiotic perspective for other neurological and psychiatric disorders in which presumed hallucinations are commonly reported (98, 99), particularly in neurodegenerative diseases (25, 100, 101).

## Hallucinations, hypnagogic/hypnopompic experiences, and lucid dreaming in Lewy body disorders

In most neurodegenerative diseases, and especially in Lewy body disorders, the evolution of the disease can produce psychotic symptoms (21), ranging from minor hallucinations (i.e. sense of presence, passage hallucinations, and visual illusions (22)) to well-structured visual hallucinations and delusions (21, 23). However, the psychotic nature of presumed hallucinations is not always clear. Patients with Lewy body disorders often also present narcolepsy-like pathology (i.e. neurodegeneration of hypocretin/orexin neurons in the hypothalamus) (102, 103) and clinical signs (24, 104), such as excessive daytime sleepiness (105) and REM-sleep behavior disorder (106). In patients with presumed hallucinations, sleep disturbances are particularly prevalent (107), and the frequency of sleep onset REM periods is higher (108), which is likely to provide privileged access to hypnagogic/hypnopompic states (100) (Fig. 1). Accordingly, presumed hallucinations are often reported to have dream-like content and to occur in situations compatible with hypnagogic/hypnopompic transitions and sleep (24–26). The widely used Parkinson's disease sleep scale (PDSS) (109) has one item that asks about “distressing hallucinations at night”, which is intended to evaluate the unclear nosological concept of “nocturnal psychosis”, well representing the ambiguity in the field.

With this dualistic clinical picture, attempts to explain presumed hallucinations in Lewy body disorders with unifying theoretical frameworks lead to incomplete if not apparently contradictory hypotheses, such as dream imagery intrusion, deficits in reality monitoring or impairment in visual perception and attention (110). However, the contradictions are perhaps not surprising nor truly contradictory, because the word “hallucinations” is being indiscriminately employed to refer to genuine psychotic hallucinations and narcolepsy-like hypnagogic/hypnopompic experiences (20, 100). Both phenomena can coexist, but they deserve to be named and considered separately (24, 111). On the one hand, genuine hallucinations provide prognostic value for faster development of cognitive impairment decline and dementia (112, 113) (hypothesis 7, Table 1), may require antipsychotic treatment, and possibly have little impact on lucid dreaming frequency. On the other hand, hypnagogic/hypnopompic experiences possibly have a more favorable prognostic value (111) (hypothesis 8, Table 1), may not require any specific pharmacological treatment, and are possibly associated with increased lucid dreaming frequency (hypothesis 9, Table 1). Importantly, genuine hallucinations and hypnagogic/hypnopompic experiences are likely driven by different pathological substrates (21, 23, 102, 103). A cleaner distinction of the two phenotypes—including their possible co-occurrence—would aid navigation through the clinical heterogeneity of Lewy body disorders and of other neurodegenerative diseases.

As potential confounding factors, dopaminergic medication—primarily employed to improve motor functions—can both worsen psychotic (or psychotic-like) symptoms (114) and cause vivid dreams, night terrors, and nightmares (115, 116), which are also suggested to predict poor motor and cognitive evolution (117, 118). Conversely, acetylcholinesterase inhibitors—primarily employed to manage cognitive impairment—stimulate lucid dreaming in healthy subjects (formally tested for galantamine) (119, 120) and often cause abnormal dreams in patients (121). Both the dopaminergic and the cholinergic systems are causally involved in the regulation of normal REM sleeping and dreaming (122–126). How many of those vivid and/or abnormal dreams may be lucid dreams remains unexplored.

Likely modulated by both disease processes and pharmacological treatments, lucid dreaming and hypnagogic/hypnopompic experiences may refine the prognostic value of presumed hallucinations for dementia (hypothesis 10, Table 1). Admittedly, this concept remains speculative at present, and its practical value, if any, will require formal investigation. A particular challenge is the efficacy and reliability of methods for independent validation of lucid dreaming, due to the inherently subjective nature of self-reports. This subjectivity may be overcome, at least in principle, with futuristic brain–computer dream reports performed in real-time while sleeping, at least to confirm lucidity (41, 127–129). Yet, the difficulty of dealing with subjective reports (i.e. symptoms) instead of objective measures (i.e. signs) is fairly common in medicine, often representing a necessary compromise in both clinical research and clinical practice (e.g. consider pain). At the very least, the dualism between genuine psychotic hallucinations and relatively normal hypnagogic/hypnopompic experiences prompts, as suggested above, for the update of clinical scales available for assessing hallucinations in order to differentiate between the two phenomena more explicitly. This differentiation would be useful not only in Lewy body disorders but also in a wide range of brain disorders that may affect—directly or iatrogenically—cortical sensory/cognitive structures and/or subcortical sleep/arousal structures, including other neurodegenerative diseases (e.g. Alzheimer's disease, vascular dementia, frontotemporal dementia, etc.) (21, 130, 131), sleep disorders (132–135), and psychiatric disorders (136, 137).

## Therapeutic value of lucid dreaming for brain disorders

A few final considerations deserve to be made about possible therapeutic implications. Lucid dreaming is often proposed to have instrumental therapeutic value, specifically as a promising approach to treat nightmare disorders (138–140) (i.e. if you give your monsters the conscious attention they are annoyingly asking for, they may tell you what they have to say and stop bothering you) (hypothesis 11, Table 1). This approach is certainly attractive for patients that achieve oneiric lucidity. Nonetheless, lucid dreaming per se may also have intrinsic therapeutic value (hypothesis 12, Table 1). Lucid dreaming is usually—although not always—rewarding, emotionally positive, and even spiritually revealing (47, 139, 141, 142). The phenomenology of lucid dreaming is similar to the experiences elicited by psychedelics (143), whose therapeutic potential has sparked renewed interest in recent years (144–146). A number of healthy people spend time, effort, and money to learn and refine the ability to lucid dream, as a nonutilitarian path toward personal fulfillment. If the positive aspects of the experience remain predominant, which is not necessarily the case, then the emergence of lucid dreaming as a consequence of a pathological process might be therapeutically valuable, at least from a psychological perspective.

For the majority of patients with aggressive neurodegenerative disorders, the idea of exploring lucid dreaming may not be particularly attractive or useful. Nevertheless, lucid dreaming and closely related hypnagogic/hypnopompic experiences exist as a continuum of normal phenomena. Some patients may become more likely to experience these phenomena and, if so, these experiences should not be considered psychotic behaviors, as they may be—or be turned into—positive experiences with possible intrinsic therapeutic value for personal fulfillment. This therapeutic value could be formally investigated with observational studies assessing the relationship between the frequency of lucid dreams (possibly stratified by emotional content) and neuropsychological scales, or with interventional studies designed to induce or facilitate lucid dreaming in normal or clinical experimental groups. These studies should also clarify the possible risks, if any, of lucid dreaming training in clinical populations. The intrinsic therapeutic potential of lucid dreaming is certainly available, at least to some patients, in narcolepsy (94, 95), possibly in Lewy body disorders (147), and other diseases, for which currently there are only anecdotal reports. The opportunity is illustratively described by a neurologist with a diagnosis of dementia with Lewy bodies, Dr. Daniel A. Drubach, who recently wrote: “Twilight [i.e. the disease] has also blessed me with lucid dreaming” (147). Importantly, lucid dreaming does not come with an instruction manual, and patients will need guidance from their doctors, who should be prepared to provide it according to the patients’ vital experiences and spiritual beliefs.

## Conclusions

**Fig. 2.**
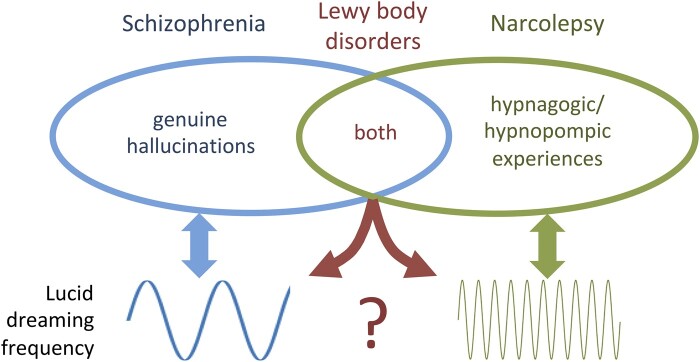
Schematic representation of the relationship between hallucinations, hypnagogic/hypnopompic experiences, and lucid dreaming in representative brain disorders. In schizophrenia, the paradigmatic disorder for hallucinations, lucid dreaming frequency is presumably normal. In narcolepsy, the paradigmatic disorder for hypnagogic/hypnopompic experiences, lucid dreaming frequency is dramatically increased. In Lewy body disorders (i.e. Parkinson's disease, Parkinson's disease dementia, and dementia with Lewy bodies), reported hallucinations are a mix of genuine psychotic hallucinations and hypnagogic/hypnopompic experiences. The frequency of lucid dreaming is expected to specifically increase with the narcolepsy-like pathology and phenotype, but this prediction awaits formal investigation.

In summary, lucid dreaming may provide conceptual and practical tools for differentiating relatively normal hypnagogic/hypnopompic experiences from psychotic hallucinations. At a conceptual level, the behavioral dynamics of wake-initiated lucid dreaming offer a possible systematization of the phenomenological range and temporal sequence of hypnagogic/hypnopompic experiences that can occur in the transition from awake to REM dreaming (including hypnagogic perceptions, transition symptoms, sleep paralysis, false awakenings, and out-of-body experiences). At a practical level, metacognitive strategies used by lucid dreamers to gain or confirm oneiric lucidity could be tested for better differentiating hallucinations from hypnagogic/hypnopompic experiences. In schizophrenia, hallucinations are paradigmatically psychotic, and lucid dreaming frequency is presumably normal. In narcolepsy, virtually all non-iatrogenic putative hallucinations are in fact hypnagogic/hypnopompic experiences, and lucid dreaming frequency is dramatically increased. In neurodegenerative diseases, putative hallucinations are a mix of genuine psychotic hallucinations and relatively normal hypnagogic/hypnopompic experiences, and the possible diagnostic, prognostic, and therapeutic value of lucid dreaming awaits formal investigation (Fig. 2). Lucid dreaming per se, if emotionally positive, may have intrinsic therapeutic value when triggered or enhanced by training/induction strategies or by a pathological process, if properly recognized and guided. This Perspective will hopefully help raise awareness and foster further research about the clinical implications of hypnagogic/hypnopompic experiences and lucid dreaming for brain disorders, for the ultimate benefit of patients.

## Data Availability

The manuscript has no associated data.

## References

[1] Schacter DL . 1976. The hypnagogic state: a critical review of the literature. Psychol Bull. 83:452–481. 778884

[2] Ghibellini R, Meier B. 2023. The hypnagogic state: a brief update. J Sleep Res. 32:e13719. 36017720 10.1111/jsr.13719 PMC10078162

[3] Stickgold R, Malia A, Maguire D, Roddenberry D, O’Connor M. 2000. Replaying the game: hypnagogic images in normals and amnesics. Science. 290:350–353. 11030656 10.1126/science.290.5490.350

[4] Rechtschaffen A, Wolpert EA, Dament WC, Mitchell SA, Fisher C. 1963. Nocturnal sleep of narcoleptics. Electroencephalogr Clin Neurophysiol. 15:599–609. 14161512 10.1016/0013-4694(63)90032-4

[5] Miyasita A, Fukuda K, Inugami M. 1989. Effects of sleep interruption on REM-NREM cycle in nocturnal human sleep. Electroencephalogr Clin Neurophysiol. 73:107–116. 2473877 10.1016/0013-4694(89)90189-2

[6] Singh M, Drake CL, Roth T. 2006. The prevalence of multiple sleep-onset REM periods in a population-based sample. Sleep. 29:890–895. 16895255 10.1093/sleep/29.7.890

[7] Bishop C, Rosenthal L, Helmus T, Roehrs T, Roth T. 1996. The frequency of multiple sleep onset REM periods among subjects with no excessive daytime sleepiness. Sleep. 19:727–730. 9122560 10.1093/sleep/19.9.727

[8] Powell AJ . 2018. Mind and spirit: hypnagogia and religious experience. Lancet Psychiatry. 5:473–475. 29628363 10.1016/S2215-0366(18)30138-X

[9] Ness RC . 1978. The old hag phenomenon as sleep paralysis: a biocultural interpretation. Cult Med Psychiatry. 2:15–39. 699620 10.1007/BF00052448

[10] Fukuda K, Miyasita A, Inugami M, Ishihara K. 1987. High prevalence of isolated sleep paralysis: kanashibari phenomenon in Japan. Sleep. 10:279–286. 3629091 10.1093/sleep/10.3.279

[11] Liddon SC . 1967. Sleep paralysis and hypnagogic hallucinations. Their relationship to the nightmare. Arch Gen Psychiatry. 17:88–96. 4378150 10.1001/archpsyc.1967.01730250090013

[12] Caraccio M, Kryger MH. 2023. Salvador Dalí: hypnagogic hallucinations in art. Sleep Health. 9:1–2. 36849219 10.1016/j.sleh.2023.01.009

[13] Siegel I, Kryger MH. 2016. When sleep is intertwined with myth. Sleep Health. 2:183–184. 29073419 10.1016/j.sleh.2016.06.006

[14] Hobson A . 2004. A model for madness? Nature. 430:21. 15229582 10.1038/430021a

[15] Iqbal N, Danish N, Ebrahim A, Naeem A, Khawaja IS. 2019. A case of narcolepsy misdiagnosed as schizophrenia. In: Khawaja I, Hurwitz T, editors. Comorbid sleep and psychiatric disorders: a clinical casebook. Cham: Springer. p. 75–83.

[16] Shapiro B, Spitz H. 1976. Problems in the differential diagnosis of narcolepsy versus schizophrenia. Am J Psychiatry. 133:1321–1323. 984224 10.1176/ajp.133.11.1321

[17] Gupta AK, Sahoo S, Grover S. 2017. Narcolepsy in adolescence-A missed diagnosis: a case report. Innov Clin Neurosci. 14:20–23. 29616151 PMC5880369

[18] Stores G . 2006. The protean manifestations of childhood narcolepsy and their misinterpretation. Dev Med Child Neurol. 48:307–310. 16542521 10.1017/S0012162206000661

[19] Douglass AB . 2003. Narcolepsy: differential diagnosis or etiology in some cases of bipolar disorder and schizophrenia? CNS Spectr. 8:120–126. 12612497 10.1017/s1092852900018344

[20] Siclari F, Valli K, Arnulf I. 2020. Dreams and nightmares in healthy adults and in patients with sleep and neurological disorders. Lancet Neurol. 19:849–859. 32949545 10.1016/S1474-4422(20)30275-1

[21] Naasan G, et al 2021. Psychosis in neurodegenerative disease: differential patterns of hallucination and delusion symptoms. Brain. 144:999–1012. 33501939 10.1093/brain/awaa413 PMC8041322

[22] Pagonabarraga J, et al 2016. Minor hallucinations occur in drug-naive Parkinson's disease patients, even from the premotor phase. Mov Disord. 31:45–52. 26408291 10.1002/mds.26432

[23] Nishio Y, et al 2017. Deconstructing psychosis and misperception symptoms in Parkinson's disease. J Neurol Neurosurg Psychiatry. 88:722–729. 28600444 10.1136/jnnp-2017-315741

[24] Ylikoski A, Martikainen K, Sarkanen T, Partinen M. 2015. Parkinson's disease and narcolepsy-like symptoms. Sleep Med. 16:540–544. 25770044 10.1016/j.sleep.2014.12.010

[25] Manni R, et al 2002. Hallucinations and sleep-wake cycle in PD: a 24-hour continuous polysomnographic study. Neurology. 59:1979–1981. 12499497 10.1212/01.wnl.0000038351.32678.4a

[26] Arnulf I, Leu-Semenescu S. 2009. Sleepiness in Parkinson's disease. Parkinsonism Relat Disord. 15:S101–S104. 10.1016/S1353-8020(09)70792-8 20082966

[27] Dijkstra N, Kok P, Fleming SM. 2022. Perceptual reality monitoring: neural mechanisms dissociating imagination from reality. Neurosci Biobehav Rev. 135:104557. 35122782 10.1016/j.neubiorev.2022.104557

[28] Dijkstra N, Fleming SM. 2023. Subjective signal strength distinguishes reality from imagination. Nat Commun. 14:1627. 36959279 10.1038/s41467-023-37322-1 PMC10036541

[29] Denis D, French CC, Gregory AM. 2018. A systematic review of variables associated with sleep paralysis. Sleep Med Rev. 38:141–157. 28735779 10.1016/j.smrv.2017.05.005

[30] Sharpless BA, Barber JP. 2011. Lifetime prevalence rates of sleep paralysis: a systematic review. Sleep Med Rev. 15:311–315. 21571556 10.1016/j.smrv.2011.01.007 PMC3156892

[31] Mainieri G, et al 2021. Are sleep paralysis and false awakenings different from REM sleep and from lucid REM sleep? A spectral EEG analysis. J Clin Sleep Med. 17:719–727. 33283752 10.5664/jcsm.9056 PMC8020694

[32] Molendijk ML, et al 2017. Prevalence rates of the Incubus phenomenon: a systematic review and meta-analysis. Front Psychiatry. 8:253. 29225584 10.3389/fpsyt.2017.00253 PMC5705555

[33] Green C . 1990. Waking dreams and other metachoric experiences. Psychiatr J Univ Ott. 15:123–128. 2374788

[34] Nielsen TA . 1991. Reality dreams and their effects on spiritual belief: a revision of animism theory. In: Gackenbach J, Sheikh A, editors. Dream images: a call to mental arms. New York (NY): Routledge.

[35] Levitan L, LaBerge S, DeGracia DJ, Zimbardo PG. 1999. Out-of-body experiences, dreams, and REM sleep. Sleep Hypn. 1:186–196.

[36] D’Agostino A, Castelnovo A, Scarone S. 2013. Dreaming and the neurobiology of self: recent advances and implications for psychiatry. Front Psychol. 4:680. 24133470 10.3389/fpsyg.2013.00680 PMC3783843

[37] Mota-Rolim SA, et al 2020. The dream of god: how do religion and science see lucid dreaming and other conscious states during sleep? Front Psychol. 11:555731. 33123040 10.3389/fpsyg.2020.555731 PMC7573223

[38] La Berge SP, Nagel LE, Dement WC, Zarcone VP. 1981. Lucid dreaming verified by volitional communication during REM sleep. Percept Mot Skills. 52:727–732. 24171230 10.2466/pms.1981.52.3.727

[39] Baird B, Mota-Rolim SA, Dresler M. 2019. The cognitive neuroscience of lucid dreaming. Neurosci Biobehav Rev. 100:305–323. 30880167 10.1016/j.neubiorev.2019.03.008 PMC6451677

[40] Voss U, et al 2014. Induction of self awareness in dreams through frontal low current stimulation of gamma activity. Nat Neurosci. 17:810–812. 24816141 10.1038/nn.3719

[41] Konkoly KR, et al 2021. Real-time dialogue between experimenters and dreamers during REM sleep. Curr Biol. 31:1417–1427.e6. 33607035 10.1016/j.cub.2021.01.026 PMC8162929

[42] LaBerge S, Baird B, Zimbardo PG. 2018. Smooth tracking of visual targets distinguishes lucid REM sleep dreaming and waking perception from imagination. Nat Commun. 9:3298. 30120229 10.1038/s41467-018-05547-0 PMC6098118

[43] Filevich E, Dresler M, Brick TR, Kühn S. 2015. Metacognitive mechanisms underlying lucid dreaming. J Neurosci. 35:1082–1088. 25609624 10.1523/JNEUROSCI.3342-14.2015 PMC6605529

[44] Dresler M, et al 2011. Dreamed movement elicits activation in the sensorimotor cortex. Curr Biol. 21:1833–1837. 22036177 10.1016/j.cub.2011.09.029

[45] Simor P, Bogdány T, Peigneux P. 2022. Predictive coding, multisensory integration, and attentional control: a multicomponent framework for lucid dreaming. Proc Natl Acad Sci U S A. 119:e2123418119. 10.1073/pnas.2123418119 PMC9636904 36279459

[46] Saunders DT, Roe CA, Smith G, Clegg H. 2016. Lucid dreaming incidence: a quality effects meta-analysis of 50 years of research. Conscious Cogn. 43:197–215. 27337287 10.1016/j.concog.2016.06.002

[47] Voss U, Schermelleh-Engel K, Windt J, Frenzel C, Hobson A. 2013. Measuring consciousness in dreams: the lucidity and consciousness in dreams scale. Conscious Cogn. 22:8–21. 23220345 10.1016/j.concog.2012.11.001

[48] Holzinger B, Mayer L. 2020. Lucid dreaming brain network based on Tholey's 7 klartraum criteria. Front Psychol. 11:1885. 32849106 10.3389/fpsyg.2020.01885 PMC7403396

[49] Baird B, Tononi G, LaBerge S. 2022. Lucid dreaming occurs in activated rapid eye movement sleep, not a mixture of sleep and wakefulness. Sleep. 45:zsab294. 10.1093/sleep/zsab294 35167686

[50] Voss U, Frenzel C, Koppehele-Gossel J, Hobson A. 2012. Lucid dreaming: an age-dependent brain dissociation. J Sleep Res. 21:634–642. 22639960 10.1111/j.1365-2869.2012.01022.x

[51] Gott J, et al 2021. Virtual reality training of lucid dreaming. Philos Trans R Soc B Biol Sci. 376:20190697. 10.1098/rstb.2019.0697 PMC7741087 33308070

[52] La Berge SP . 1980. Lucid dreaming as a learnable skill: a case study. Percept Mot Skills. 51:1039–1042.

[53] Tan S, Fan J. 2023. A systematic review of new empirical data on lucid dream induction techniques. J Sleep Res. 32:e13786. 36408823 10.1111/jsr.13786

[54] Laberge S, Levitan L, Dement WC. 1986. Lucid dreaming: physiological correlates of consciousness during REM sleep. J Mind Behav. 7:251–258.

[55] Stumbrys T, Erlacher D, Schädlich M, Schredl M. 2012. Induction of lucid dreams: a systematic review of evidence. Conscious Cogn. 21:1456–1475. 22841958 10.1016/j.concog.2012.07.003

[56] Waters F, et al 2016. What is the link between hallucinations, dreams, and hypnagogic–hypnopompic experiences? Schizophr Bull. 42:1098–1109. 27358492 10.1093/schbul/sbw076 PMC4988750

[57] Ro T, Ellmore TM, Beauchamp MS. 2013. A neural link between feeling and hearing. Cereb Cortex. 23:1724–1730. 22693344 10.1093/cercor/bhs166 PMC3673182

[58] Liang M, Mouraux A, Hu L, Iannetti GD. 2013. Primary sensory cortices contain distinguishable spatial patterns of activity for each sense. Nat Commun. 4:1979. 23752667 10.1038/ncomms2979 PMC3709474

[59] Pérez-Bellido A, Anne Barnes K, Crommett LE, Yau JM. 2018. Auditory frequency representations in human somatosensory cortex. Cereb Cortex. 28:3908–3921. 29045579 10.1093/cercor/bhx255 PMC6188539

[60] Sharpless BA . 2014. Exploding head syndrome. Sleep Med Rev. 18:489–493. 24703829 10.1016/j.smrv.2014.03.001

[61] American Academy of Sleep Medicine . 2014. International classification of sleep disorders. 3rd ed. Darien (IL): American Academy of Sleep Medicine.

[62] Dahlitz M, Parkes JD. 1993. Sleep paralysis. Lancet. 341:406–407. 8094172 10.1016/0140-6736(93)92992-3

[63] Takeuchi T, Miyasita A, Sasaki Y, Inugami M, Fukuda K. 1992. Isolated sleep paralysis elicited by sleep interruption. Sleep. 15:217–225. 1621022 10.1093/sleep/15.3.217

[64] Buzzi G . 2019. False awakenings in lucid dreamers: how they relate with lucid dreams, and how lucid dreamers relate with them. Dreaming. 29:323–338.

[65] Voss U, Holzmann R, Tuin I, Hobson JA. 2009. Lucid dreaming: a state of consciousness with features of both waking and non-lucid dreaming. Sleep. 32:1191–1200. 19750924 10.1093/sleep/32.9.1191 PMC2737577

[66] Takeuchi T, Miyasita A, Inugami M, Sasaki Y, Fukuda K. 1994. Laboratory-documented hallucination during sleep-onset REM period in a normal subject. Percept Mot Skills. 78:979–985. 8084722 10.1177/003151259407800355

[67] Kliková M, Sharpless BA, Bušková J. 2021. Could sleep paralysis be pleasant? J Sleep Res. 30:e13154. 32869388 10.1111/jsr.13154

[68] Herrero NL, Gallo FT, Gasca-Rolín M, Gleiser PM, Forcato C. 2023. Spontaneous and induced out-of-body experiences during sleep paralysis: emotions, “AURA” recognition, and clinical implications. J Sleep Res. 32:e13703. 36053735 10.1111/jsr.13703

[69] Mahowald MW, Schenck CH. 1992. Dissociated states of wakefulness and sleep. Neurology. 42:44–51. discussion 52. 1630638

[70] Tong F . 2003. Out-of-body experiences: from Penfield to present. Trends Cogn Sci. 7:104–106. 12639686 10.1016/s1364-6613(03)00027-5

[71] Blanke O, Landis T, Spinelli L, Seeck M. 2004. Out-of-body experience and autoscopy of neurological origin. Brain. 127:243–258. 14662516 10.1093/brain/awh040

[72] De Ridder D, Van Laere K, Dupont P, Menovsky T, Van de Heyning P. 2007. Visualizing out-of-body experience in the brain. N Engl J Med. 357:1829–1833. 17978291 10.1056/NEJMoa070010

[73] Blanke O, Ortigue S, Landis T, Seeck M. 2002. Stimulating illusory own-body perceptions. Nature. 419:269–270. 12239558 10.1038/419269a

[74] Raduga M, Kuyava O, Sevcenko N. 2020. Is there a relation among REM sleep dissociated phenomena, like lucid dreaming, sleep paralysis, out-of-body experiences, and false awakening? Med Hypotheses. 144:110169. 32795836 10.1016/j.mehy.2020.110169

[75] Denis D, Poerio GL. 2017. Terror and bliss? Commonalities and distinctions between sleep paralysis, lucid dreaming, and their associations with waking life experiences. J Sleep Res. 26:38–47. 27460633 10.1111/jsr.12441 PMC5245115

[76] Mallett R, Sowin L, Raider R, Konkoly KR, Paller KA. 2022. Benefits and concerns of seeking and experiencing lucid dreams: benefits are tied to successful induction and dream control. Sleep Adv. 3:zpac027. 10.1093/sleepadvances/zpac027 PMC10104404 37193400

[77] Vallat R, Ruby PM. 2019. Is it a good idea to cultivate lucid dreaming? Front Psychol. 10:2585. 31803118 10.3389/fpsyg.2019.02585 PMC6874013

[78] Roberts DM, Schade MM, Mathew GM, Gartenberg D, Buxton OM. 2020. Detecting sleep using heart rate and motion data from multisensor consumer-grade wearables, relative to wrist actigraphy and polysomnography. Sleep. 43:zsaa045. 32215550 10.1093/sleep/zsaa045 PMC7355403

[79] Mueser KT, Bellack AS, Brady EU. 1990. Hallucinations in schizophrenia. Acta Psychiatr Scand. 82:26–29. 2399817 10.1111/j.1600-0447.1990.tb01350.x

[80] Dresler M, et al 2015. Neural correlates of insight in dreaming and psychosis. Sleep Med Rev. 20:92–99. 25092021 10.1016/j.smrv.2014.06.004

[81] Voss U, et al 2018. Insight and dissociation in lucid dreaming and psychosis. Front Psychol. 9:2164. 30483185 10.3389/fpsyg.2018.02164 PMC6241172

[82] Mota NB, Resende A, Mota-Rolim SA, Copelli M, Ribeiro S. 2016. Psychosis and the control of lucid dreaming. Front Psychol. 7:294. 27014118 10.3389/fpsyg.2016.00294 PMC4783408

[83] David AS, Bedford N, Wiffen B, Gilleen J. 2012. Failures of metacognition and lack of insight in neuropsychiatric disorders. Philos Trans R Soc B Biol Sci. 367:1379–1390. 10.1098/rstb.2012.0002 PMC3318769 22492754

[84] Bassetti CLA, et al 2019. Narcolepsy—clinical spectrum, aetiopathophysiology, diagnosis and treatment. Nat Rev Neurol. 15:519–539. 31324898 10.1038/s41582-019-0226-9

[85] Nishino S, Ripley B, Overeem S, Lammers GJ, Mignot E. 2000. Hypocretin (orexin) deficiency in human narcolepsy. Lancet. 355:39–40. 10615891 10.1016/S0140-6736(99)05582-8

[86] Thannickal TC, et al 2000. Reduced number of hypocretin neurons in human narcolepsy. Neuron. 27:469–474. 11055430 10.1016/s0896-6273(00)00058-1 PMC8760623

[87] Feng H, et al 2020. Orexin signaling modulates synchronized excitation in the sublaterodorsal tegmental nucleus to stabilize REM sleep. Nat Commun. 11:4190. 32694504 10.1038/s41467-020-17401-3 PMC7374574

[88] Hanin C, et al 2021. Narcolepsy and psychosis: a systematic review. Acta Psychiatr Scand. 144:28–41. 33779983 10.1111/acps.13300 PMC8360149

[89] Fortuyn HAD, et al 2009. Psychotic symptoms in narcolepsy: phenomenology and a comparison with schizophrenia. Gen Hosp Psychiatry. 31:146–154. 19269535 10.1016/j.genhosppsych.2008.12.002

[90] Douglass AB, Hays P, Pazderka F, Russell JM. 1991. Florid refractory schizophrenias that turn out to be treatable variants of HLA-associated narcolepsy. J Nerv Ment Dis. 179:12–17. 1985143 10.1097/00005053-199101000-00003

[91] Douglass AB, et al 1993. Schizophrenia, narcolepsy, and HLA-DR15, DQ6. Biol Psychiatry. 34:773–780. 8292681 10.1016/0006-3223(93)90066-m

[92] Andlauer O, et al 2013. Nocturnal rapid eye movement sleep latency for identifying patients with narcolepsy/hypocretin deficiency. JAMA Neurol. 70:891. 23649748 10.1001/jamaneurol.2013.1589 PMC4170796

[93] Gott J, et al 2020. Sleep fragmentation and lucid dreaming. Conscious Cogn. 84:102988. 32768920 10.1016/j.concog.2020.102988

[94] Dodet P, Chavez M, Leu-Semenescu S, Golmard J-L, Arnulf I. 2015. Lucid dreaming in narcolepsy. Sleep. 38:487–497. 25348131 10.5665/sleep.4516 PMC4335518

[95] Rak M, Beitinger P, Steiger A, Schredl M, Dresler M. 2015. Increased lucid dreaming frequency in narcolepsy. Sleep. 38:787–792. 25325481 10.5665/sleep.4676 PMC4402667

[96] Wamsley E, Donjacour CEHM, Scammell TE, Lammers GJ, Stickgold R. 2014. Delusional confusion of dreaming and reality in narcolepsy. Sleep. 37:419–422. 24501437 10.5665/sleep.3428 PMC3900627

[97] Lacaux C, et al 2019. Increased creative thinking in narcolepsy. Brain. 142:1988–1999. 31143939 10.1093/brain/awz137

[98] Manford M . 1998. Complex visual hallucinations. Clinical and neurobiological insights. Brain. 121:1819–1840. 9798740 10.1093/brain/121.10.1819

[99] Fraser CL, Lueck CJ. 2021. Illusions, hallucinations, and visual snow. Handb Clin Neurol. 178:311–335. 33832684 10.1016/B978-0-12-821377-3.00014-3

[100] Arnulf I, et al 2000. Hallucinations, REM sleep, and Parkinson's disease. Neurology. 55:281–288. 10908906 10.1212/wnl.55.2.281

[101] Leu-Semenescu S, et al 2011. Hallucinations in narcolepsy with and without cataplexy: contrasts with Parkinson's disease. Sleep Med. 12:497–504. 21486708 10.1016/j.sleep.2011.03.006

[102] Fronczek R, et al 2007. Hypocretin (orexin) loss in Parkinson's disease. Brain. 130:1577–1585. 17470494 10.1093/brain/awm090

[103] Thannickal TC, Lai Y-Y, Siegel JM. 2007. Hypocretin (orexin) cell loss in Parkinson's disease. Brain. 130:1586–1595. 17491094 10.1093/brain/awm097 PMC8762453

[104] Arnulf I . 2005. Excessive daytime sleepiness in parkinsonism. Sleep Med Rev. 9:185–200. 15893249 10.1016/j.smrv.2005.01.001

[105] Feng F, et al 2021. Excessive daytime sleepiness in Parkinson's disease: a systematic review and meta-analysis. Parkinsonism Relat Disord. 85:133–140. 33637423 10.1016/j.parkreldis.2021.02.016

[106] Blesa J, Foffani G, Dehay B, Bezard E, Obeso JA. 2022. Motor and non-motor circuit disturbances in early Parkinson disease: which happens first? Nat Rev Neurosci. 23:115–128. 34907352 10.1038/s41583-021-00542-9

[107] Kulisevsky J, Roldan E. 2004. Hallucinations and sleep disturbances in Parkinson's disease. Neurology. 63:S28–S30. 10.1212/wnl.63.8_suppl_3.s28 15505140

[108] Nomura T, et al 2003. Visual hallucinations as REM sleep behavior disorders in patients with Parkinson's disease. Mov Disord. 18:812–817. 12815661 10.1002/mds.10439

[109] Chaudhuri KR . 2002. The Parkinson's disease sleep scale: a new instrument for assessing sleep and nocturnal disability in Parkinson's disease. J Neurol Neurosurg Psychiatry. 73:629–635. 12438461 10.1136/jnnp.73.6.629 PMC1757333

[110] Muller AJ, Shine JM, Halliday GM, Lewis SJG. 2014. Visual hallucinations in Parkinson's disease: theoretical models. Mov Disord. 29:1591–1598. 25154807 10.1002/mds.26004

[111] Omoto S, et al 2021. Risk factors for minor hallucinations in Parkinson's disease. Acta Neurol Scand. 143:538–544. 33222164 10.1111/ane.13380

[112] Marinus J, Zhu K, Marras C, Aarsland D, van Hilten JJ. 2018. Risk factors for non-motor symptoms in Parkinson's disease. Lancet Neurol. 17:559–568. 29699914 10.1016/S1474-4422(18)30127-3

[113] Aarsland D, et al 2021. Parkinson disease-associated cognitive impairment. Nat Rev Dis Prim. 7:47. 34210995 10.1038/s41572-021-00280-3

[114] Cummings JL . 1991. Behavioral complications of drug treatment of Parkinson's disease. J Am Geriatr Soc. 39:708–716. 2061539 10.1111/j.1532-5415.1991.tb03627.x

[115] Sharf B, Moskovitz C, Lupton MD, Klawans HL. 1978. Dream phenomena induced by chronic levodopa therapy. J Neural Transm. 43:143–151. 104005 10.1007/BF01579073

[116] Pinter MM, Pogarell O, Oertel WH. 1999. Efficacy, safety, and tolerance of the non-ergoline dopamine agonist pramipexole in the treatment of advanced Parkinson's disease: a double blind, placebo controlled, randomised, multicentre study. J Neurol Neurosurg Psychiatry. 66:436–441. 10201413 10.1136/jnnp.66.4.436 PMC1736320

[117] Otaiku AI . 2021. Dream content predicts motor and cognitive decline in Parkinson's disease. Mov Disord Clin Pract. 8:1041–1051. 34631940 10.1002/mdc3.13318 PMC8485616

[118] Bugalho P, et al 2021. Do dreams tell the future? Dream content as a predictor of cognitive deterioration in Parkinson's disease. J Sleep Res. 30:e13163. 32776436 10.1111/jsr.13163

[119] LaBerge S, LaMarca K, Baird B. 2018. Pre-sleep treatment with galantamine stimulates lucid dreaming: a double-blind, placebo-controlled, crossover study. PLoS One. 13:e0201246. 30089135 10.1371/journal.pone.0201246 PMC6082533

[120] Sparrow G, Hurd R, Carlson R, Molina A. 2018. Exploring the effects of galantamine paired with meditation and dream reliving on recalled dreams: toward an integrated protocol for lucid dream induction and nightmare resolution. Conscious Cogn. 63:74–88. 29960246 10.1016/j.concog.2018.05.012

[121] Russ TC, Morling JR. 2012. Cholinesterase inhibitors for mild cognitive impairment. Cochrane Database Syst Rev. 2016:CD009132. 10.1002/14651858.CD009132.pub2 PMC6464825 22972133

[122] Hasegawa E, et al 2022. Rapid eye movement sleep is initiated by basolateral amygdala dopamine signaling in mice. Science. 375:994–1000. 35239361 10.1126/science.abl6618

[123] Van Dort CJ, et al 2015. Optogenetic activation of cholinergic neurons in the PPT or LDT induces REM sleep. Proc Natl Acad Sci U S A. 112:584–589. 25548191 10.1073/pnas.1423136112 PMC4299243

[124] Sarter M, Bruno J. 1999. Cortical cholinergic inputs mediating arousal, attentional processing and dreaming: differential afferent regulation of the basal forebrain by telencephalic and brainstem afferents. Neuroscience. 95:933–952. 10.1016/s0306-4522(99)00487-x 10682701

[125] Solms M . 2000. Dreaming and REM sleep are controlled by different brain mechanisms. Behav Brain Sci. 23:843–850. 11515144 10.1017/s0140525x00003988

[126] Kumar Yadav R, Mallick BN. 2021. Dopaminergic- and cholinergic-inputs from substantia nigra and pedunculo-pontine tegmentum, respectively, converge in amygdala to modulate rapid eye movement sleep in rats. Neuropharmacology. 193:108607. 34023337 10.1016/j.neuropharm.2021.108607

[127] Raduga M . 2021. Detecting lucid dreams only by submentalis electromyography. Sleep Med. 88:221–230. 34798438 10.1016/j.sleep.2021.10.030

[128] Raduga M . 2022. “I love you”: the first phrase detected from dreams. Sleep Sci. 15:149–157. 10.5935/1984-0063.20220035 PMC9210561 35755904

[129] Mallett R . 2020. A pilot investigation into brain-computer interface use during a lucid dream. Int J Dream Res. 13:62–69.

[130] Hampel H, et al 2018. The cholinergic system in the pathophysiology and treatment of Alzheimer's disease. Brain. 141:1917–1933. 29850777 10.1093/brain/awy132 PMC6022632

[131] Martorana A, Koch G. 2014. Is dopamine involved in Alzheimer's disease? Front Aging Neurosci. 6:252. 25309431 10.3389/fnagi.2014.00252 PMC4174765

[132] Aldrich MS . 1991. The neurobiology of narcolepsy. Trends Neurosci. 14:235–239. 1716016 10.1016/0166-2236(91)90121-a

[133] Mignot E, Taheri S, Nishino S. 2002. Sleeping with the hypothalamus: emerging therapeutic targets for sleep disorders. Nat Neurosci. 5(Suppl):1071–1075. 12403989 10.1038/nn944

[134] Sani OG, et al 2018. Mood variations decoded from multi-site intracranial human brain activity. Nat Biotechnol. 36:954–961. 30199076 10.1038/nbt.4200

[135] Yang Y, et al 2021. Mesencephalic dopamine neurons are essential for modafinil-induced arousal. Br J Pharmacol. 178:4808–4825. 34399438 10.1111/bph.15660

[136] Joshi YB, et al 2021. Anticholinergic medication burden–associated cognitive impairment in schizophrenia. Am J Psychiatry. 178:838–847. 33985348 10.1176/appi.ajp.2020.20081212 PMC8440496

[137] Lieberman JA, First MB. 2018. Psychotic disorders. N Engl J Med. 379:270–280. 30021088 10.1056/NEJMra1801490

[138] de Macêdo TCF, Ferreira GH, de Almondes KM, Kirov R, Mota-Rolim SA. 2019. My rules: can lucid dreaming treat nightmares? Front Psychol. 10:2618. 31849749 10.3389/fpsyg.2019.02618 PMC6902039

[139] Holzinger B, Klösch G, Saletu B. 2015. Studies with lucid dreaming as add-on therapy to gestalt therapy. Acta Neurol Scand. 131:355–363. 25639732 10.1111/ane.12362

[140] Ouchene R, El Habchi N, Demina A, Petit B, Trojak B. 2023. The effectiveness of lucid dreaming therapy in patients with nightmares: a systematic review. Encephale. 49:525–531. 37005191 10.1016/j.encep.2023.01.008

[141] Stocks A, et al 2020. Dream lucidity is associated with positive waking mood. Conscious Cogn. 83:102971. 32535498 10.1016/j.concog.2020.102971

[142] Charlton BG . 2007. Alienation, recovered animism, and altered states of consciousness. Med Hypotheses. 68:727–731. 17141965 10.1016/j.mehy.2006.11.004

[143] Sanz C, Zamberlan F, Erowid E, Erowid F, Tagliazucchi E. 2018. The experience elicited by hallucinogens presents the highest similarity to dreaming within a large database of psychoactive substance reports. Front Neurosci. 12:7. 29403350 10.3389/fnins.2018.00007 PMC5786560

[144] Griffiths RR, et al 2016. Psilocybin produces substantial and sustained decreases in depression and anxiety in patients with life-threatening cancer: a randomized double-blind trial. J Psychopharmacol. 30:1181–1197. 27909165 10.1177/0269881116675513 PMC5367557

[145] Carhart-Harris R, et al 2021. Trial of psilocybin versus escitalopram for depression. N Engl J Med. 384:1402–1411. 33852780 10.1056/NEJMoa2032994

[146] dos Santos RG, Hallak JEC. 2020. Therapeutic use of serotoninergic hallucinogens: a review of the evidence and of the biological and psychological mechanisms. Neurosci Biobehav Rev. 108:423–434. 31809772 10.1016/j.neubiorev.2019.12.001

[147] Jaffee MS, Drubach DA. 2021. Twilight and me: a soliloquy. Continuum (Minneap Minn). 27:1809–1817. 34881738 10.1212/CON.0000000000001091

